# Netherton Syndrome in Thai Children: A Report of Two Cases With a Literature Review

**DOI:** 10.7759/cureus.62718

**Published:** 2024-06-19

**Authors:** Panipak Temboonnark, Tassalapa Daengsuwan

**Affiliations:** 1 Division of Allergy, Immunology, and Rheumatology, Department of Pediatrics, Queen Sirikit National Institute of Child Health, Bangkok, THA; 2 College of Medicine, Rangsit University, Bangkok, THA

**Keywords:** atopic dermatitis, congenital ichthyosis, trichorrhexis invaginata, atopic diathesis, netherton syndrome

## Abstract

Netherton syndrome (NS) is a severe autosomal recessive disorder characterized by the triad of congenital ichthyosiform erythroderma, trichorrhexis invaginata, and atopic diathesis. We report two cases that experienced severe congenital exfoliative dermatitis, recurrent infections, and allergic conditions. Examinations of hair under the light microscope revealed trichorrhexis invaginata. Whole exome sequencing identified homologous pathogenic mutations of SPINK5. Optimal skincare and proper nutritional support could improve patients’ quality of life.

## Introduction

Netherton syndrome (NS) is a rare autosomal recessive disorder with an incidence of approximately one in 200,000 [1]. NS is characterized by the triad of congenital ichthyosiform erythroderma or ichthyosis linearis circumflexa, trichorrhexis invaginata (bamboo hair), and atopic diathesis (elevate serum IgE) [2,3].

Here, we report two cases of NS, who presented with congenital exfoliative dermatitis and recurrent infections in early life.

## Case presentation

Case 1

A five-month-old Thai girl presented at our hospital with exfoliative dermatitis. She was born at 37 weeks of gestation to non-consanguineous parents. Shortly after birth, she had congenital pneumonia and developed exfoliative dermatitis on her face, trunk, and extremities. She had a history of severe infections, including three episodes of septicemia, three cases of pneumonia, one instance of acute bilateral otitis media, infective endocarditis, and subdural empyema. *Staphylococcus aureus*, *Acinetobacter baumannii*, and *Enterococcus faecalis* were identified from her blood cultures.

**Figure 1:**
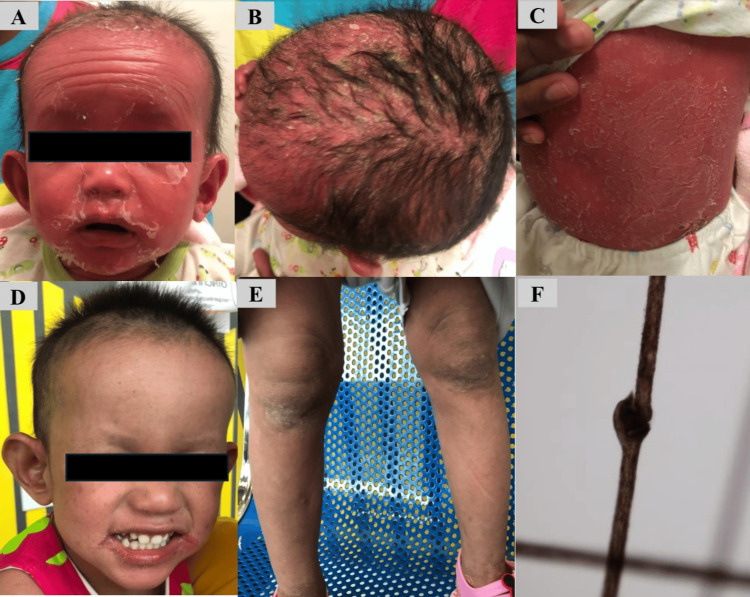
Case 1 was pictured at six months old (A, B, C) and three years old (D, E). Trichorrhexis invaginata presented in case 1 (F).

The physical examination at five months old revealed a female infant with generalized erythroderma. Her weight and height were below the third percentile. The skin of her face, trunk, and extremities was shiny, thick, and scaly. Her hair and eyebrows were sparse, while her nails and mucosae were normal (Figure 1A-1C).

Regarding recurrent severe infections, especially the exploration of serious uncommon organisms, such as *Acinetobacter baumannii* and *Enterococcus faecalis*, which were not usually found in healthy children, and failure to thrive led us to investigate the immunocompromise tendency in this patient. However, there were no abnormal levels of lymphocyte count, IgG, IgA, IgM, and lymphocyte subset counts (CD3+, CD4+, CD8+, CD19+, and CD16+56+), apart from the elevation of serum total IgE 1,070 IU/ml (normal 0.19-16.9 IU/ml) (Table 1). The specific IgE for cow’s milk and soy were 1.250 and 0.04 KUA/L, respectively (normal < 0.35 KUA/L). Cow’s milk protein allergy was suspected. Consequently, she was switched from cow’s milk formula to amino acid formula and received treatment with emollients, but her skin condition did not improve. A skin biopsy was taken from her thigh. Histological examination demonstrated that the epidermis showed focal parakeratosis, focal spongiosis with lymphocytic exocytosis, and irregular acanthosis. The dermis showed dilated blood vessels, mild fibrosis, and perivascular lymphohistiocytic inflammation. The pathological diagnosis of a skin biopsy was consistent with exfoliative dermatitis. However, the examination of her hair under light microscopy revealed swelling and invagination of the hair shaft (Figure 1F). We conducted genetic testing by extracting DNA from her blood and subsequently performed whole exome sequencing using the Agilent Sure Select Human All Exon V6kit capture kit. All rare alleles were analyzed for pathogenicity, confirming the diagnosis of Netherton syndrome through the identification of a homozygous NM_001127698.1ex8.649C>T,p.Arg217*(rs367958902) mutation in the SPINK5 gene. She received aggressive treatment with emollients and a one-month course of therapy with an oral retinoid at 0.5 mg/kg/day. Her skin gradually improved after stepping down to cow’s milk-based formula. She had never got any infection after six months of age. 

**Table 1:** Laboratory investigations in case 1 WBC, white cell count; ALC, absolute lymphocyte count; AEC, absolute eosinophil count; CD, cluster of designation; IgM, immunoglobulin M; IgA, immunoglobulin A; IgG, immunoglobulin G; IgE, immunoglobulin E

Investigations	Value	Reference range	Unit
WBC	13390	5200-11000	cells/μL
ALC	5757	2300-5400	cells/μL
AEC	0	<500	cells/μL
Lymphocyte subpopulation			
CD3+ T cells	4143	1400-3700	cells/μL
%CD3+ T cells	60.3	28-75	%
CD4+ T cells	2061	700-2200	cells/μL
%CD4+ T cells	30.0	28-47	%
CD8+ T cells	1727	490-1300	cells/μL
%CD8+ T cells	25.1	16-30	%
CD19+ B cells	2215	390-1400	cells/μL
%CD19+ B cells	30.8	14-33	%
CD16+56+ NK cells	541	130-720	cells/μL
%CD16+56+ NK cells	7.5	4-17	%
Immunoglobulin			
IgM	88.5	47-200	mg/dL
IgA	98.5	22-159	mg/dL
IgG	1260	441-1135	mg/dL
IgE	1070	0.19-16.9	IU/mL
Specific IgE			
Cow’s milk	1.25	< 0.35	KUA/L
Soy	0.04	< 0.35	KUA/L

At a three-year follow-up visit, her skin condition significantly improved, and her nutritional status became normal. She had generalized mild erythroderma, whereas desquamated skin was found only on the dorsal side of both hands (Figure 1D, 1E). Her body weight and height were within a normal range. We are continuing to follow up with the patient. 

Case 2

**Figure 2:**
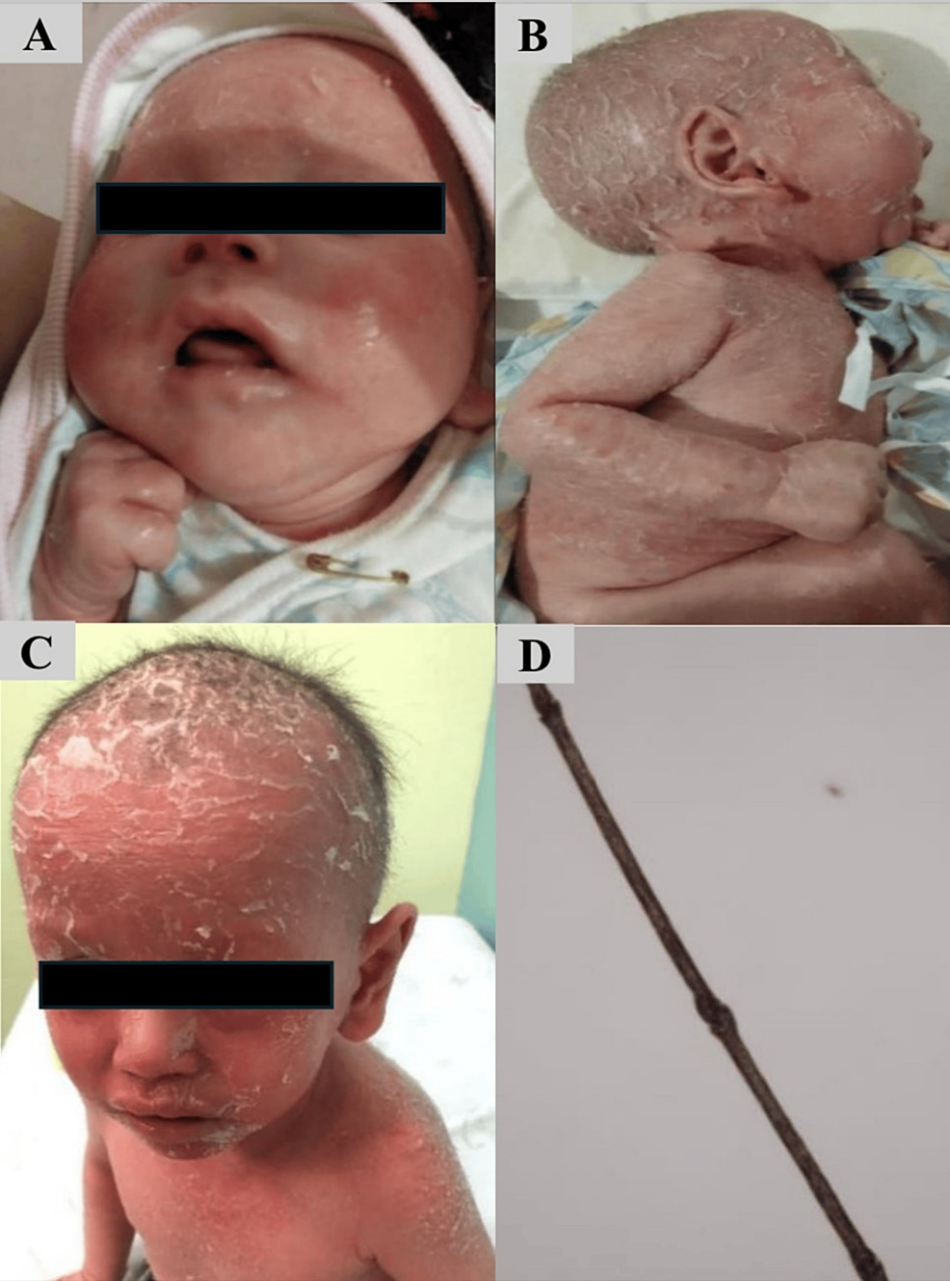
Case 2 was pictured at two months old (A, B) and three years old (C). Trichorrhexis invaginata presented in case 2 (D).

A two-month-old Thai boy was referred to our hospital due to severe congenital exfoliative dermatitis. Physical examinations revealed generalized scaling erythroderma on the face, trunk, and extremities. His hair and eyebrows were sparse and fragile (Figure 2A, 2B).

He was born at 34 weeks gestation with a birth weight of 2,625 grams to non-consanguineous parents. He was documented to have generalized erythroderma and desquamation since the first day of life. The first infection, which occurred when he was seven months old, was acute bilateral otitis media. In the first two years of his life, he experienced recurrent ear infections 10 times, including acute otitis media and acute otitis externa, as well as two skin infections. The most severe infection was *Staphylococcus epidermidis* septicemia when he was one and a half years old. At nine months old, a skin biopsy was performed due to his severe exfoliative skin. The results showed parakeratosis, neutrophil collection in the stratum corneum, and irregular acanthosis with rete ridge elongation in the epidermis. There were dilated capillaries and superficial perivascular lymphohistiocytic infiltration in the upper dermis. This result was compatible with psoriasis. However, the examination of his hair under a light microscope showed bamboo hair, meaning the distal part invaginated into the proximal portion of the hair shaft (Figure 2D). Finally, whole exome sequencing revealed homologous splicing variants NM_001127698:exon24:C.2313+1G>C pathogenic mutation in the SPINK5 gene, confirming the diagnosis of NS diagnosis.

The patient’s complete blood counts revealed leukocytosis and eosinophilia. The immunologic evaluation at two years old was normal (Table 2). However, IgE was high at 4,180 IU/mL (normal 0.31-29.5 IU/ml). Serum phytohemagglutinin showed normal activity.

**Table 2:** Laboratory investigations in case 2 WBC, white cell count; ALC, absolute lymphocyte count; AEC, absolute eosinophil count; CD, cluster of designation; IgM, immunoglobulin M; IgA, immunoglobulin A; IgG, immunoglobulin G; IgE, immunoglobulin E

Investigations	Value	Reference range	Unit
WBC	24770	6400-12000	cells/μL
ALC	9660	3600-5400	cells/μL
AEC	1734	<500	cells/μL
Lymphocyte subpopulation			
CD3+ T cells	6000	2100-6200	cells/μL
%CD3+ T cells	51.9	53-75	%
CD4+ T cells	2900	1300-3400	cells/μL
%CD4+ T cells	24.7	32-51	%
CD8+ T cells	2900	620-2000	cells/μL
%CD8+ T cells	22.5	14-30	%
CD19+ B cells	4700	720-2600	cells/μL
%CD19+ B cells	38.1	16-35	%
CD20+ B cells	4700	-	cells/μL
%CD20+ B cells	38.2	-	%
CD16+56+ NK cells	1300	180-920	cells/μL
%CD16+56+ NK cells	10.6	3-15	%
Immunoglobulin			
IgM	53.3	47-200	mg/dL
IgA	74.2	22-159	mg/dL
IgG	786	441-1135	mg/dL
IgE	4180	0.31-29.5	IU/mL
Specific IgE			
Cow’s milk	5.54	<0.35	KUA/L
Soy	11.40	<0.35	KUA/L
Egg white	26.90	<0.35	KUA/L
Egg yolk	3.23	<0.35	KUA/L
Wheat	8.83	<0.35	KUA/L

In addition, his skin worsened after consuming cow’s milk and wheat. Therefore, he was evaluated for food allergy when he was two years old. His food-specific IgE concentrations were positive (Table 2). Thus, his mother was advised to avoid feeding him cow’s milk, soy, egg white, egg yolk, and wheat products and to treat exfoliative skin with moisturizers, acitretin, and 1% hydrocortisone. Currently, he is three years old and continues to avoid the aforementioned allergenic foods. His skin condition is improved (Figure 2C). He has lived without any infections since he was two years old.

## Discussion

NS is caused by germline mutations in the SPINK5 (serine protease inhibitor, Kazal type-5) gene located on chromosome 5q31-32 [4,5]. SPINK5 encoded protein named LEKTI (lympho-epithelial Kazal-type related inhibitor), which is exceedingly expressed in hair follicles, epithelia, and thymus [6,7]. LEKTI is a marker of normal differentiated human primary keratinocytes [8]. Consequently, SPINK5 mutations caused the premature desquamation of the stratum corneum [9]. In addition, LEKTI has a key role in anti-inflammatory and anti-microbial mechanisms in mucous epithelia [1]. Besides skin, LEKTI is also highly expressed in the thymus, where T-cell maturation, selection, and antigen processing develop. Then, the defect of LEKTI expression leads to tissue inflammation and overexpression of Th2 and Th17 responses [1,2,10].

NS is typically characterized by the triad of ichthyosis linearis circumflexa, trichorrhexis invaginate, and atopic diathesis [3,11]. Affected neonates present with congenital ichthyosiform erythroderma, characterized by generalized erythroderma and desquamation. In severe patients, the erythroderma may turn to migratory, serpiginous, erythematous plaques with double-edged scales (ichthyosis linearis circumflexa) [12]. The skin conditions can cause life-threatening complications, including severe dehydration, hypernatremia, and sepsis, specifically in newborns [12]. Both of our patients presented with infantile exfoliative erythroderma. They were initially diagnosed with severe atopic dermatitis, which is significantly more prevalent than NS. However, when other clinical manifestations, including thin hair and recurrent infection, occurred, NS was suspected. The hairs and eyebrows are frequently sparse and dysplastic in NS. Trichorrhexis invaginate (bamboo hair) is a pathognomonic sign of NS and is characterized by an invagination of the shaft's distal portion to the proximal portion owing to the defect in the keratinization in the inner root sheath. This hair abnormality, examined under light microscopy or trichoscopy, can be found in scalp hair, eyebrows, and eyelashes [2,3,11,13]. Our patients’ hair examination revealed trichorrhexis invaginate; therefore, a provisional diagnosis of NS was made in both children.

Even though NS is a skin disorder, a skin biopsy could not be the diagnosing tool because the histopathology was nonspecific [11]. Histological features from skin biopsies of patients with NS are primarily defined as psoriasiform hyperplasia [14,15]. The skin biopsy of our patients also showed epidermal parakeratosis, compatible with psoriasis and exfoliative dermatitis. The gold standard for definite diagnosis of NS is the identification of pathologic variants in SPINK5 gene, which are found in both patients [1,12]. Allergic conditions are other common manifestations of NS [2,6]. Even though our patients showed extremely high levels of total IgE, only case 2 had an allergic problem, which was multiple food allergies. Case 1 had no allergic manifestation until the age of 3. Nonetheless, we continue to follow both patients.

NS is now categorized as a primary immunodeficiency disorder, specifically a syndrome with immunodeficiency. Immunologic disorders are associated with B-cell defects, including increased IgE levels, hypogammaglobulinemia, and impaired antibody response to pneumococcal immunization, whereas T cells are normal in number and function [16,17]. Our patients had a history of recurrent sinopulmonary tract infections, which were possible due to B-cell disorder. However, basic immunologic evaluations were normal in both patients. Although their immune responses to pneumococcal vaccines were not assessed, our cases were administered the pneumococcal vaccines to prevent pneumococcal diseases in the future.

Furthermore, the first and second patients had a history of septicemia due to *Staphylococcus aureus* and *Staphylococcus epidermidis*, respectively. These bacteria are commonly found on the human skin. Poor skin integrity allows the microorganism to penetrate the skin to blood steam. After improving dermatologic conditions, optimizing nutritional support, and appropriate immunization, both patients had no severe or recurrent infections.

To our knowledge, there is no specific treatment for NS. Eczema and desquamation, especially in the neonatal period, require extensive skincare to protect the skin barrier and diminish complications [9,18]. The current guidelines for managing congenital ichthyoses suggest using topical agents, such as emollients, keratolytics, and antiseptics, as first-line treatments [19].

Topical corticosteroids, such as hydrocortisone, triamcinolone acetonide, and mometasone furoate, as well as topical calcineurin inhibitors, including 0.03% and 0.1% tacrolimus ointment (Protopic®), manufactured by Astellas Pharma Co., Japan, and 1% pimecrolimus cream (Elidel®), manufactured by Bausch Health Companies Inc., Canada, are commonly used for a range of skin conditions and can alleviate the dermatological symptoms of NS. However, both agents were recommended for intermittent utilization under monitoring their side effects [6,13]. Our patients were mainly treated with moisturizers and intermittent topical corticosteroids. Their skin conditions gradually improved.

Systemic therapy should be considered in patients with NS who have severe skin conditions and fail to respond to traditional treatments. A systematic review demonstrated that dupilumab, a fully human monoclonal antibody blocking interleukin (IL)-4 and IL-13, and secukinumab, an IL-17 antagonist, effectively control dermatologic symptoms in NS. The dosing regimens for dupilumab and secukinumab were determined according to clinical trials or guideline recommendations for atopic dermatitis and psoriasis, respectively. Dupilumab treatment was initiated subcutaneously at 600 mg, followed by a maintenance dose of 300 mg administered every two weeks in adults. Pediatric patients received dupilumab dosing adjusted based on age and weight, following a clinical trial or a national treatment guideline for atopic dermatitis. Secukinumab was administered subcutaneously using a weight-adjusted dosing regimen: 75 mg for less than 25 kg, 150 mg for 25 to 50 kg, and 300 mg for greater than 50 kg at baseline and at weeks 1, 2, 3, 4, and subsequently monthly [20]. Advanced understanding of the disease mechanism could lead to more effective targeted therapies in the future, which could improve the quality of life in patients with NS [6].

## Conclusions

We reported two infants presented with congenital ichthyosiform erythroderma, trichorrhexis invaginata, and atopic diathesis. Molecular studies confirmed the SPINK5 mutation, the pathogenic gene mutation in NS. Providing adequate skincare and proper nutritional support could improve patients’ quality of life. Further basic and clinical research is required to provide NS's effective or specific treatment.
